# Performance and clinical utility of supervised machine-learning approaches in detecting familial hypercholesterolaemia in primary care

**DOI:** 10.1038/s41746-020-00349-5

**Published:** 2020-10-30

**Authors:** Ralph K. Akyea, Nadeem Qureshi, Joe Kai, Stephen F. Weng

**Affiliations:** Primary Care Stratified Medicine, Division of Primary Care, University of Nottingham, Nottingham, UK

**Keywords:** Dyslipidaemias, Outcomes research

## Abstract

Familial hypercholesterolaemia (FH) is a common inherited disorder, causing lifelong elevated low-density lipoprotein cholesterol (LDL-C). Most individuals with FH remain undiagnosed, precluding opportunities to prevent premature heart disease and death. Some machine-learning approaches improve detection of FH in electronic health records, though clinical impact is under-explored. We assessed performance of an array of machine-learning approaches for enhancing detection of FH, and their clinical utility, within a large primary care population. A retrospective cohort study was done using routine primary care clinical records of 4,027,775 individuals from the United Kingdom with total cholesterol measured from 1 January 1999 to 25 June 2019. Predictive accuracy of five common machine-learning algorithms (logistic regression, random forest, gradient boosting machines, neural networks and ensemble learning) were assessed for detecting FH. Predictive accuracy was assessed by area under the receiver operating curves (AUC) and expected vs observed calibration slope; with clinical utility assessed by expected case-review workload and likelihood ratios. There were 7928 incident diagnoses of FH. In addition to known clinical features of FH (raised total cholesterol or LDL-C and family history of premature coronary heart disease), machine-learning (ML) algorithms identified features such as raised triglycerides which reduced the likelihood of FH. Apart from logistic regression (AUC, 0.81), all four other ML approaches had similarly high predictive accuracy (AUC > 0.89). Calibration slope ranged from 0.997 for gradient boosting machines to 1.857 for logistic regression. Among those screened, high probability cases requiring clinical review varied from 0.73% using ensemble learning to 10.16% using deep learning, but with positive predictive values of 15.5% and 2.8% respectively. Ensemble learning exhibited a dominant positive likelihood ratio (45.5) compared to all other ML models (7.0–14.4). Machine-learning models show similar high accuracy in detecting FH, offering opportunities to increase diagnosis. However, the clinical case-finding workload required for yield of cases will differ substantially between models.

## Introduction

Familial hypercholesterolaemia (FH) is a common inherited genetic disorder causing high cholesterol levels from birth^1^ and increased risk of premature heart disease and death^2^. FH affects ~1 in 200–500 of the general population^3,4^. However, most individuals with FH and affected family members remain undiagnosed worldwide^5^. In patients with heterozygous FH, lipid-lowering therapy such as the use of moderate- to high-intensity statins, or newer PCSK9 inhibitors markedly improves prognosis^6^ – reducing risk of coronary heart disease and all-cause mortality by at least 44%^7,8^. Patients who remain unidentified will be untreated or be sub-optimally treated with low-intensity statins and assumed to have commoner multifactorial causes for raised cholesterol.

Internationally, current approaches to identity FH based on clinical characteristics recommend use of the Simon-Broome diagnostic criteria (SB)^2^, Dutch Lipid Clinic Network criteria (DLCN)^9^, Make Early Diagnosis to Prevent Early Deaths (MEDPED)^10^, or Japanese Atherosclerosis Society (JAS) criteria^11^ (see Supplementary Box 1). These criteria have all been developed from specialist FH or lipid clinic registries, with emphasise on conducting a thorough family history and assessment of clinical features such as tendon xanthoma and arcus senilis. This means the application of these criteria in searching electronic health records of the wider general population in primary care will be limited. For instance, family histories are poorly recorded as evidenced in primary care databases from the UK and Australia, hence an acknowledged limitation of using primary care databases^12^.

Hence, there has been a drive to develop bespoke algorithms derived from large electronic health records (EHRs) to detect FH. The SEARCH study in the US^13^ used an electronic version of the DLCN criteria, while the FAMCAT tool in the UK^14–16^ and FindFH model in the US^17^ have been recently developed from prediction modelling. Using standard logistic regression, area under receiver operating curves (AUC) for FAMCAT were from 0.86 in its development database (UK Clinical Practice Research Datalink)^18^, to 0.83 and 0.84 in two separate external validation databases^14,15^ (QRESEARCH and RCGP Surveillance Network). Developed as a data-driven machine-learning (ML) algorithm from US administrative health data, the recent FindFH model resulted in an AUC of 0.89 using a random forest models approach^17^.

ML algorithms have diverse applications including disease modelling^19^, with the potential of improving prediction, identifying latent variables which are unlikely to be observed but might be inferred from other variables. ML, therefore, offers an alternative approach to standard prediction modelling^20^. The aims of this study were firstly, to evaluate the performance of a range of different ML algorithms to identify patients with FH within a large UK general primary care population; secondly, we sought to determine potential differences in the clinical utility of using different ML algorithms.

## Results

### Study population characteristics

**Fig. 1:**
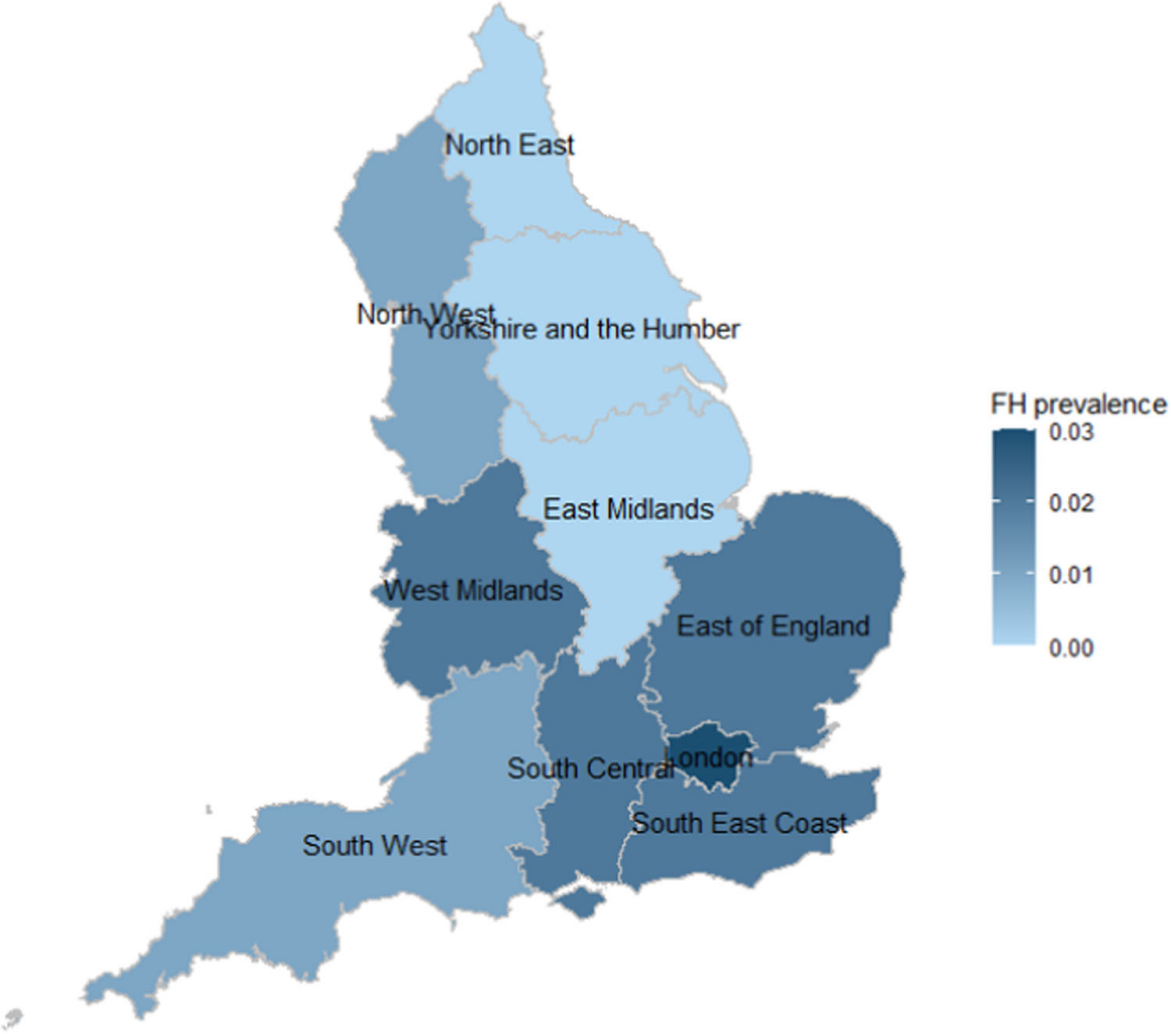
Map of familial hypercholesterolaemia prevalence. Reported prevalence of familial hypercholesterolaemia in primary care electronic health records by English region.

There was a total of 4,157,705 individuals in CPRD study population with either a total cholesterol or LDL-cholesterol record during the study period. 129,930 (3.1%) individuals were excluded from the analysis for either having outlying cholesterol measurements, data entry errors, having a death or transfer out date before study start date or a diagnosis of FH before the study start date. The complete study cohort for analysis was made up of 4,027,775 individuals, with 7928 (0.2%) having a documented diagnosis of FH (Fig. 1). Reported FH prevalence was higher towards the south of England, with London having greatest frequency of FH identified. Other regions of England towards the North and Northeast have lower population frequency of FH identified.

To develop the FH models, 75% of the complete cohort (*n* = 3,020,832) was randomly sampled to become the training cohort and the remaining 25% of the cohort (*n* = 1,006,943) assigned as the validation cohort. Table 1 shows the descriptive characteristics of both training and validation cohorts stratified by sex.

**Table 1:** Clinical characteristics for men and women age 16 years or above in the derivation and validation cohorts.

	Training cohort (*n* = 3,020,832)		Validation cohort (*n* = 1,006,943)	
	Men	Women	Men	Women
Total sample size	1,464,128 (48.5)	1,556,704 (51.5)	487,071 (48.4)	519,872 (51.6)
No (%) diagnosed with FH	2527 (0.2)	3452 (0.2)	817 (0.2)	1132 (0.2)
Highest total cholesterol (TC) ever, mean (SD)	5.6 (1.3)	5.8 (1.4)	5.6 (1.3)	5.8 (1.4)
Age at TC measurement, mean (SD)	55.3 (15.8)	57.0 (17.3)	55.3 (15.8)	57.0 (17.3)
Triglyceride at time of highest TC, mean (SD)	1.9 (1.6)	1.5 (1.0)	1.9 (1.6)	1.5 (1.1)
Diastolic blood pressure at highest TC, mean (SD)	81 (11)	79 (11)	81 (11)	79 (11)
Systolic blood pressure at highest TC, mean (SD)	137 (19)	134 (20)	137 (19)	134 (20)
Highest LDL cholesterol (LDL-C) ever, mean (SD)	3.5 (1.0)	3.5 (1.1)	3.5 (1.0)	3.5 (1.1)
Age at LDL-C measurement, mean (SD)	56.1 (16.6)	57.2 (18.1)	56.2 (16.6)	57.2 (18.1)
Triglyceride at time of highest LDL-C, mean (SD)	1.7 (1.1)	1.4 (0.8)	1.7 (1.1)	1.4 (0.9)
Diastolic blood pressure at highest LDL-C, mean (SD)	81 (11)	78 (11)	81 (11)	78 (11)
Systolic blood pressure at highest LDL-C, mean (SD)	136 (18)	133 (20)	136 (18)	132 (20)
Tendon xanthomata	35 (<0.01)	56 (<0.01)	15 (<0.01)	19 (<0.01)
Family history of FH	5522 (0.4)	8852 (0.6)	1847 (0.4)	2935 (0.6)
Family history of CHD	53,105 (3.6)	69,806 (4.5)	17,675 (3.6)	23,219 (4.5)
Family history of raised cholesterol	7602 (0.5)	10,485 (0.7)	2513 (0.5)	3570 (0.7)
Family history of MI	43,620 (3.0)	47,839 (3.1)	14,742 (3.0)	16,158 (3.1)
Body mass index, mean (SD)	28.0 (4.7)	27.8 (5.9)	28.0 (4.7)	27.8 (5.9)
Smoking status				
Non-smoker	705,878 (48.2)	956,996 (61.5)	234,004 (48.0)	319,413 (61.4)
Ex-smoker	491,858 (33.6)	376,326 (24.2)	163,994 (33.7)	125,919 (24.2)
Current smoker	266,392 (18.2)	223,382 (14.4)	89,073 (18.3)	74,540 (14.3)
Alcohol status				
Non-drinker	418,657 (28.6)	603,787 (38.8)	139,229 (28.6)	201,069 (38.7)
Ex-drinker	55,943 (3.8)	46,680 (3.0)	18,392 (3.8)	15,578 (3.0)
Drinks alcohol	989,528 (67.6)	906,237 (58.2)	329,450 (67.6)	303,225 (58.3)
Hypertension	355,388 (24.3)	378,706 (24.3)	118,645 (24.4)	126,557 (24.3)
Chronic kidney disease	145,606 (9.9)	193,754 (12.5)	48,508 (9.9)	64,300 (12.4)
Hypothyroidism	42,382 (2.9)	155,282 (9.9)	13,859 (2.9)	51,770 (9.9)
Diabetes	218,271 (14.9)	179,788 (11.6)	72,671 (14.9)	59,968 (11.5)
Chronic liver disease	36,603 (2.5)	31,508 (2.0)	12,087 (2.5)	10,426 (2.0)
Nephrotic syndrome	1807 (0.1)	1396 (0.1)	562 (0.1)	482 (0.1)
History of CHD	179,494 (12.3)	114,314 (7.3)	59,814 (12.3)	38,228 (7.4)
History of CVA	44,819 (3.1)	44,171 (2.8)	14,907 (3.1)	14,764 (2.8)
History of PVD	24,130 (1.7)	15,998 (1.0)	7887 (1.6)	5253 (1.0)
Lipid-lowering medication				
No statin prescription	869,788 (59.4)	1,047,007 (67.3)	288,997 (59.3)	349,298 (67.2)
Low potency statin	31,422 (2.2)	29,592 (1.9)	10,543 (2.2)	10,014 (1.9)
Medium potency statin	332,747 (22.7)	292,403 (18.8)	111,313 (22.9)	97,552 (18.8)
High potency statin	225,434 (15.4)	180,912 (11.6)	74,602 (15.3)	60,669 (11.7)
Other lipid-lowering drugs	4737 (0.3)	6790 (0.4)	1616 (0.3)	2339 (0.5)

Family history of CHD excludes myocardial infarction. Statins were grouped into three different intensity categories according to the percentage reduction in low-density lipoprotein cholesterol^28^, based on UK National Institute for Health and Care Excellence (NICE) Clinical Guideline (CG 181).

*CHD* coronary heart disease, *CVA* cerebrovascular accident, *FH* familial hypercholesterolaemia, *PVD* peripheral vascular disease, *SD* standard deviation.

### Variable rankings

**Fig. 2:**
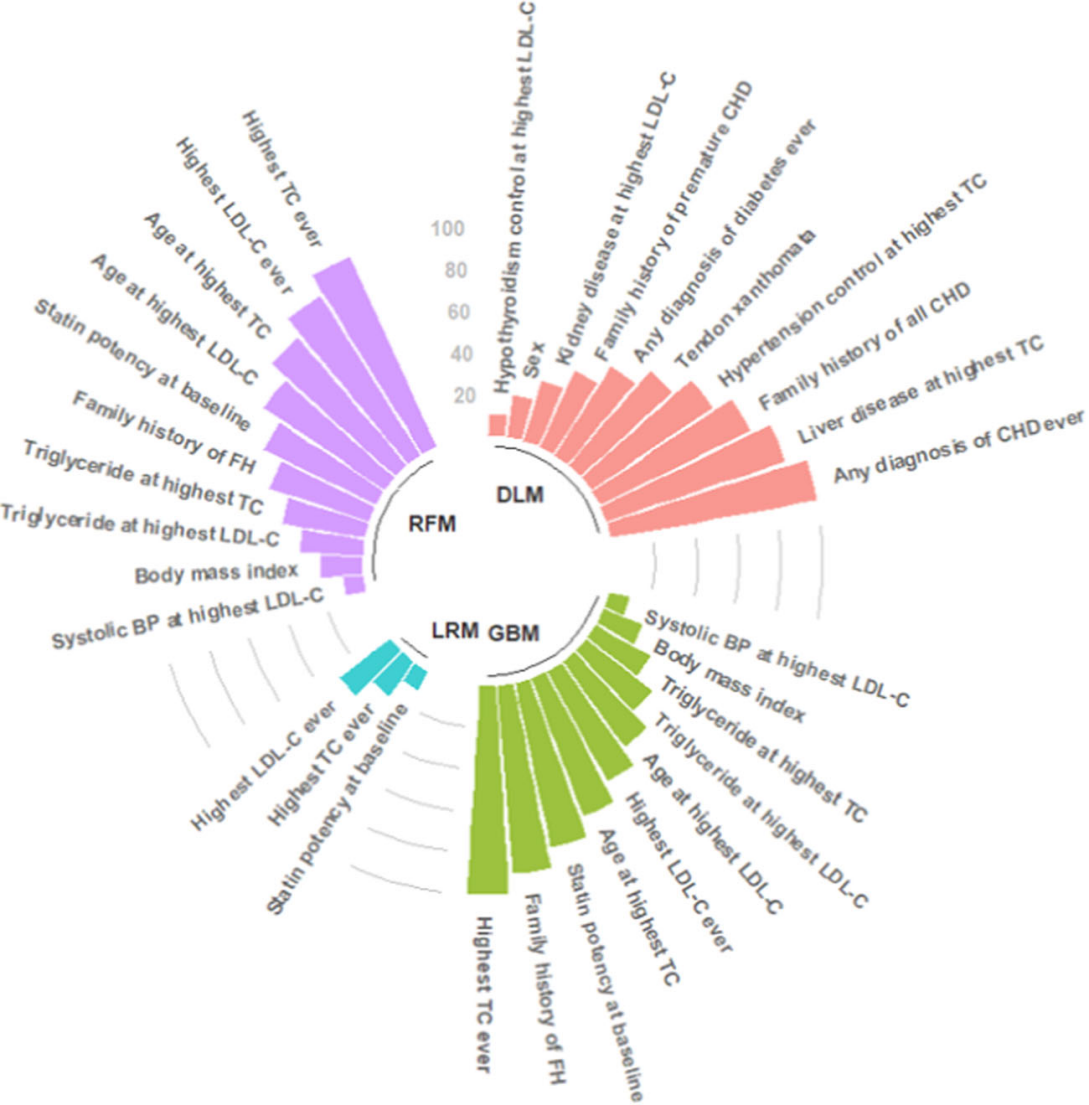
Top 10 risk factors for familial hypercholesterolaemia. Algorithms were derived from training cohort of 3,020,832 patients. BP blood pressure, CHD coronary heart disease, DLM deep learning model, FH familial hypercholesterolaemia, GBM gradient boosting model, LDL-C low-density lipoprotein cholesterol, LRM logistic regression model, RFM random forest model, TC total cholesterol.

Clinical features ranking for predicting FH are presented in Fig. 2, Supplementary Table 1. All 45 predictor variables were included in developing all the models. All models, apart from deep learning, indicated that cholesterol values and family history features were strong indicators of FH, consistent with existing diagnostic criteria. For the logistic regression model, only three features remained as relevant. For random forests and gradient boosting machines both featured current statin potency, triglycerides, body mass index and systolic blood pressure to determine the likelihood of FH. The deep learning model prioritised exclusion features which indicate a lower likelihood of FH, including secondary causes of raised cholesterol due to chronic conditions such as kidney disease, diabetes, hypertension and hypothyroidism. The deep learning model also identified rare signals such as tendon xanthomata, which is known to be under-recognised in primary care.

### Discrimination

**Fig. 3:**
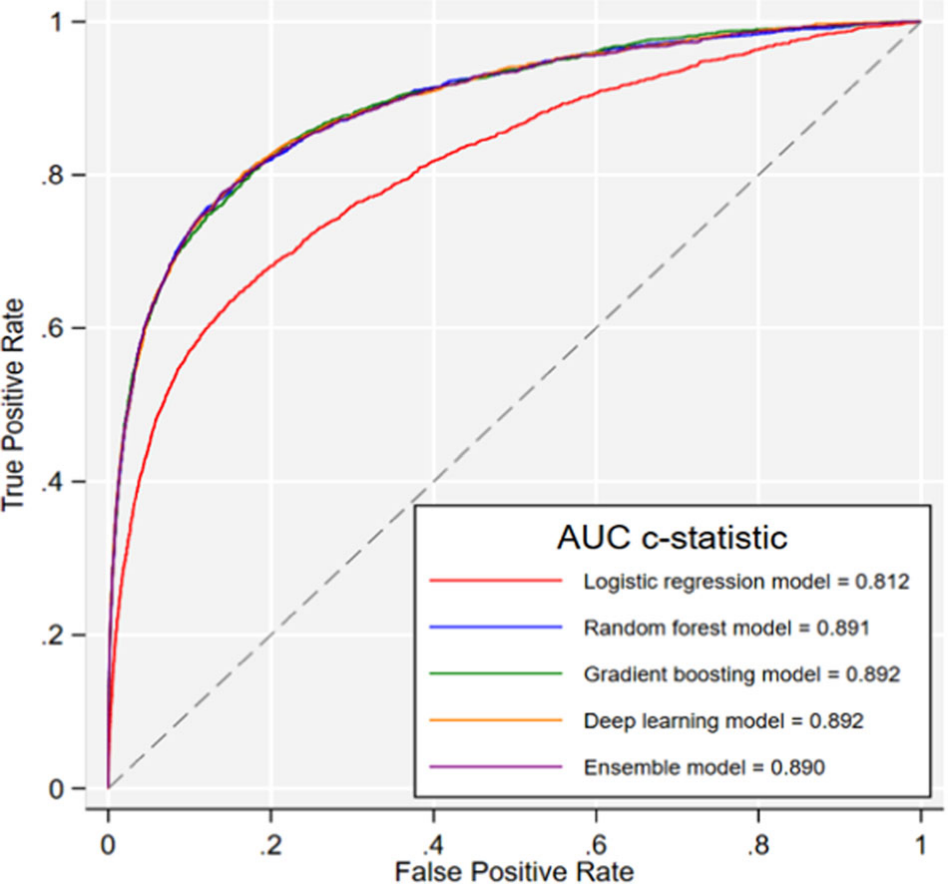
Discrimination accuracy of the models for identifying familial hypercholesterolaemia in primary care. Based on validation cohort of 1,006,943 patients.

To predict the risk/probability of having FH for each individual, the algorithms were applied to the validation cohort (*n* = 1,006,943). The discrimination accuracy based on AUC, c-statistics, is presented in Fig. 3 for all the models (Supplementary Table 2 for details). AUC was lowest for the logistic regression model. The discrimination accuracy was similar by sex. For instance, for the ensemble model, which is a combination of all the other models, the c-statistics for FH in men was 0.898 (95% CI: 0.886–0.911) compared to 0.884 (95% CI: 0.873–0.895) for women.

### Calibration

**Fig. 4:**
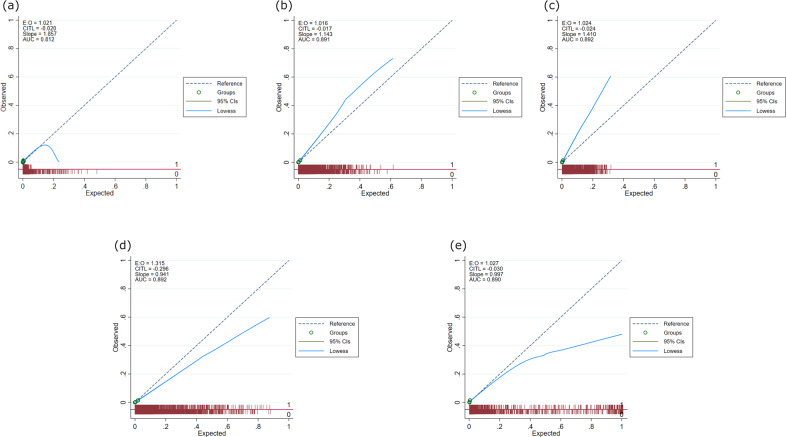
Calibration plots for all models. Based on validation cohort of 1,006,943 patients: **a** logistic regression model, **b** random forest model, **c** gradient boosting model, **d** deep-learning model and **e** ensemble model. E:O log of the expected/observed number of events, CITL calibration-in-the-large, AUC area under the curve, slope calibration slope. The circles represent deciles of patients grouped by similar predicted risk. The distribution of patients (stratified by outcome) is indicated with spikes at the bottom of the graph. Patients with a diagnosis of familial hypercholesterolaemia (FH) are represented by spikes above the *x*-axis (red line), and patients without a diagnosis of FH, below the *x*-axis).

Calibration accuracy for all the models was assessed by plotting deciles of predicted risk against expected proportion of FH diagnosis for each decile, Fig. 4. At lower predicted risks, the algorithms were generally well calibrated, however, at higher predicted risks were not as well-calibrated.

### Sensitivity and specificity

Using a cut-off above 1 in 250 (0·004), we determined the sensitivity, specificity, positive predictive value (PPV) and negative predictive value (NPV) of each machine-learning algorithm shown in Table 2. The number of high probability individuals varied due to the shapes of the probability distributions between algorithms, ranging from 0.73% of the population using ensemble learning to 10.16% of the population using deep learning. Specificity was not as variable as sensitivity ranging from 90.0% for deep leaning to 99.3% for ensemble learning. The corresponding NPVs for all algorithms were all above 99%. Sensitivity was highest for the deep learning algorithm (72.6%) and lowest for ensemble learning (30.5%). However, a lower proportion of high probability individuals using ensemble learning models meant that the PPV for ensemble learning would be the highest (15.5%). In contrast, deep learning models, by identifying a far greater number of high probability individuals, meant that this model would result in the lowest PPV (2.8%).

**Table 2:** Sensitivity, specificity, positive predictive and negative values for machine-learning models for detecting familial hypercholesterolaemia in the validation cohort (*n* = 1,006,943).

Machine-learning models	% High probability (>1/250) (%)	Sensitivity	Specificity	Positive predictive value (PPV)	Negative predictive value (NPV)
Logistic regression	3.38	37.6% (35.5–39.8)	96.7% (96.6–96.7)	4.4% (4.1–4.6)	99.7% (99.7–99.8)
Random forest	8.09	69.1% (67.0–71.2)	92.0% (92.0–92.1)	3.4% (3.3–3.5)	99.9% (99.9–99.9)
Gradient boosting	4.27	58.3% (56.1–60.5)	95.8% (95.8–95.9)	5.3% (5.1–5.5)	99.8% (99.8–99.8)
Deep learning	10.16	72.6% (70.6–74.6)	90.0% (89.9–90.0)	2.8% (2.8–2.9)	99.9% (99.9–99.9)
Ensemble learning	0.73	30.5% (28.4–32.6)	99.3% (99.3–99.3)	15.5% (14.5–16.4)	99.7% (99.7–99.7)

^a^Assumes population frequency of familial hypercholesterolaemia of 1 in 250^4^.

### Likelihood ratios

**Fig. 5:**
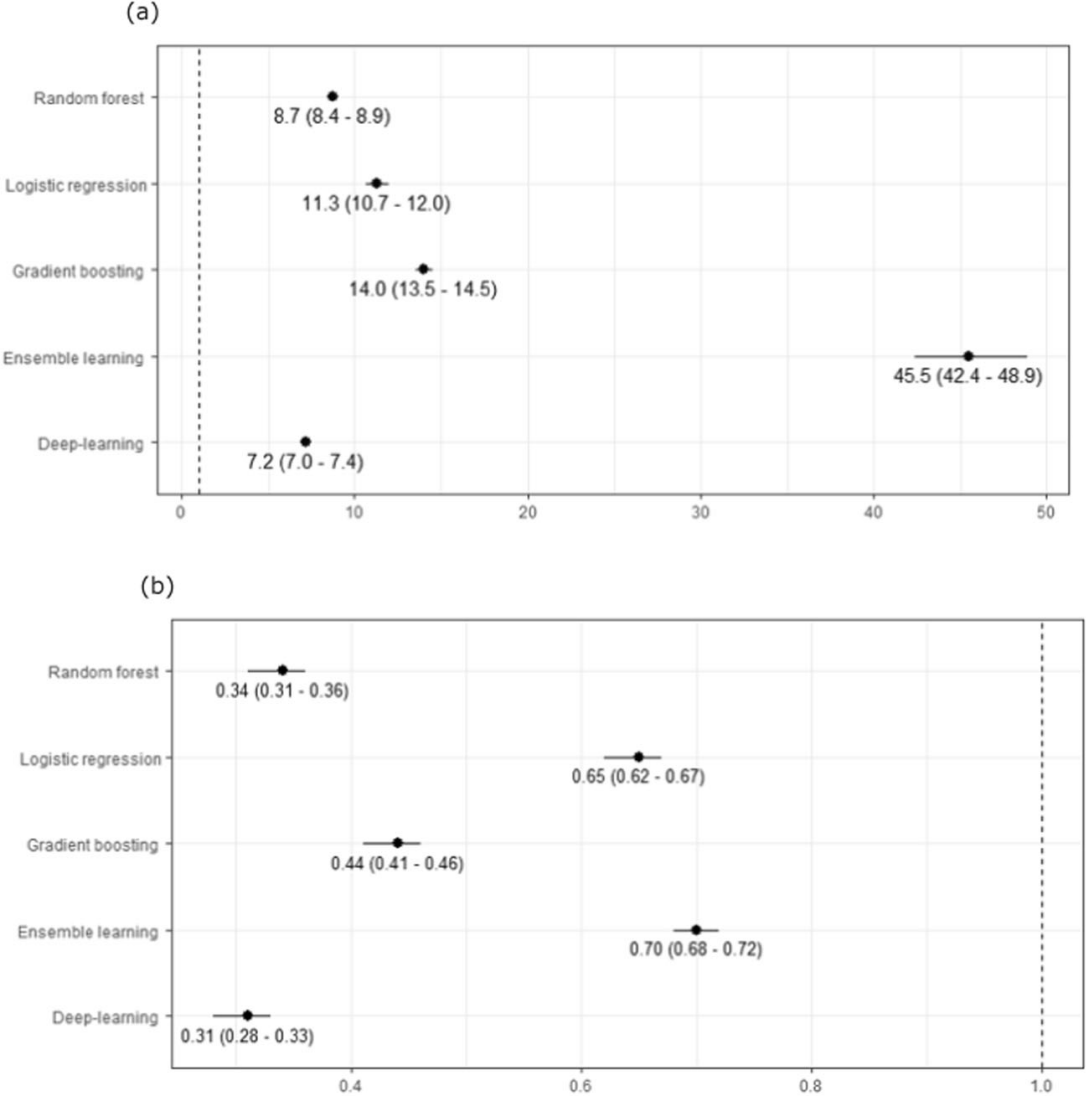
Positive and negative likelihood ratios for machine-learning models for detecting familial hypercholesterolaemia. Based on validation cohort of 1,006,943 patients: **a** positive likelihood ratios (95% confidence interval) and **b** negative likelihood ratios (95% confidence interval).

We determined positive and negative likelihood ratios (LR) for each machine-learning algorithm (Fig. 5). The positive likelihood ratio (LR+) estimates the likelihood of having FH, give a positive test result (>1 in 250). The negative likelihood ratio (LR−) estimates the likelihood of not having FH, given a negative test result (≤1/250). All machine-learning models resulted in significant LR+ and LR−, with ensemble learning having the highest LR+ (45.5, 95% CI 42.4–49.9) and deep learning models having lowest LR− (0.31, 95% CI 0.28–0.33).

## Discussion

We have assessed the ability of five different machine-learning (ML) algorithms to detect cases of familial hypercholesterolaemia (FH) in over 4 million patients’ routine primary health care records.

We found four ML models (random forest, gradient boosting, deep learning and ensemble learning) all had similarly high predictive accuracy, with AUC > 0.89. This is highly consistent with that found for the FindFH model, using a random forest algorithm in US administrative health data^17^; and a 3–6% improvement on the UK FAMCAT tool using standard logistic regression^14,15,18^.

We found substantial differences for clinical utility between ML algorithms. Despite their similar overall accuracy (apart from logistic regression), our analysis highlights a trade-off that will be necessary between specificity and sensitivity of these models for binary risk stratification. Specificity and negative predictive values were consistently high across all methods, due to the low prevalence of FH in the general population. However, numbers identified as high probability of FH, sensitivity and positive predictive values varied between approaches.

For instance, we found a deep learning model would identify ~10% of the population as probable FH, generating a very high case load for clinicians to screen, review and test. This would have the highest sensitivity (i.e. proportion of patients with actual FH identified) but the detection rate would be poor (low positive predictive value). Conversely, ensemble learning would identify only 0.73% of the population as probable FH requiring clinical review. Although this has lower sensitivity, it would be more efficient in having a higher detection rate (higher positive predictive value).

For example, in an average sized UK primary care practice of 8800 patients, an estimated 30% (*n* = 2640) of individuals would have had a registered cholesterol measured^21^. Using a deep learning model would identify 264 probable FH needing clinical assessment and testing. This strategy would yield the maximum absolute number of FH cases identified but would also be the most resource intensive. Conversely, ensemble learning would minimise the number of probable FH to <20 patients for clinical assessment. As the ensemble learning algorithm has the greatest positive likelihood ratio whilst maintaining a significant negative likelihood ratio, this may arguably be the most viable ML model to implement for FH case-finding in primary care practice, given workload and resource implications. Following a model-based approach to case-finding for potential FH, the patient would require a detailed clinical assessment and confirmatory diagnosis by genetic testing to identify a pathogenic mutation. Hence, the extent of false-positive results would have significant resource implications.

This study further highlights interesting and significant variations in the clinical variables identified by the different ML models used. The ML-based logistic regression only consisted of three variables (total cholesterol, LDL-cholesterol and potency of statin prescribed) which is similar to the initial triage of primary care electronic health records recommended by English NICE guideline recommendations through identification of elevated cholesterols alone to systematically identify those with possible FH^22^. Other models also identified potential negative indicators of FH – those with elevated triglycerides and secondary causes of raised cholesterol. In FH patients, serum triglycerides are usually not elevated^23^. Raised triglycerides appeared as an important negative feature in both random forest and gradient boosting models. Deep learning identified several secondary causes which were strong negative indicators of FH, including liver disease, chronic kidney disease, hypothyroidism and diabetes. These factors are supported by guidelines recommending excluding these secondary causes prior to establishing a possible FH diagnosis^22^. The deep learning model also identified tendon xanthomata as an important clinical feature suggesting a definite FH diagnosis, in line with established SB and DLCN criteria^2,9^. Given its poor recognition in primary care, in any standard modelling, this would have been very unlikely to have the statistical power to identify FH given this clinical feature is only present in <0.01% of the total cohort population.

This research offers a number of strengths. Our study evaluates a range of different ML models for detection of FH in primary care and has done so using not only conventional AUC but also the sensitivity, specificity, positive predictive value and negative predictive values. We employed a large sample size of over four million patients, embracing 6% of the entire UK population, enhancing generalisability of the findings. In particular, this work has also assessed the clinical value of these ML algorithms by exploring diagnostic test accuracy metrics, seldom reported for prediction models using machine-learning.

The current UK study and recent study in the US^17^ confirm that ML approaches are viable to use in EHR systems and can significantly enhance detection FH. This offers major opportunities to increase diagnosis of FH and to prevent premature heart disease and early deaths. Moreover, while replication of ML methods can be questioned^24^, the use of different datasets in the UK and US, with consistency between their analysis by different study teams is now available, supporting the generalisability of these ML approaches. In this regard, we have also made fully available, in GitHub, the codes for our models to assist with replication, validation and implementation.

However, we acknowledge several study limitations, in common with other research using large health care databases. These include lack of formal adjudication of diagnoses, information bias and potential bias due to missing data. Missing data could potentially introduce bias in the effect estimates of the prediction models as well as a reducing power. However, we used imputation methods for variables which were sufficiently missing-at-random and a very large sample size to mitigate these effects. The specific coding of FH recorded in UK general practice records will include patients identified with phenotypic FH, who may or may not have been confirmed by genetic testing. A recognised issue in EHRs is that some patients FH could potentially be misclassified, have not yet been identified, or might not have had cholesterol assessed.

Future research should validate and replicate our ML models in other large clinical datasets in other populations. Secondly, further evaluation of the feasibility and acceptability of machine-learning applications in clinical practice is needed. The computational capacity of health care systems continues to evolve; and electronic health records are increasingly moving to cloud-based servers with data centralisation. This presents exciting opportunities to exploit machine-learning as a realistic option to detect uncommon conditions of major health importance, such as familial hypercholesterolaemia.

## Methods

### Study design and data source

The study cohort was obtained from the Clinical Practice Research Datalink (CPRD). CPRD contains anonymised electronic medical records from 836 general practices with over 11 million research-usable patients^25^ and is representative of the UK general population^26^. Over 5.2 million of these patient records are currently of active research-quality, making CPRD one of the most widely used real-world data sources for healthcare research. Information routinely collected from primary care practices, as part of the database, include demographics, lifestyle, diagnoses, prescriptions, diagnostic tests, referrals to specialists and secondary care and death status. Secondary care activity is incorporated in the primary care records through hospital discharge letters from hospital or referral notes from specialists.

### Study population

A record of cholesterol level is essential to establish a diagnosis of FH hence, all patients included in the study had at least a single record of total cholesterol measurement between the baseline date of 1 January 1999 or the earliest date the CPRD primary care practice started contributing data to the database after 1 January 1999 and the end date of 25 June 2019 or the latest date the CPRD primary care practice finished contributing data prior to 25 June 2019. Where follow-up was not completed by a patient, the end date was specified as the date of death, transfer out of practice, or final practice visit. Patients with a diagnosis of FH, had the date of the diagnosis specified as their end date. Patients aged 16 years and younger were excluded as the cholesterol level thresholds for the diagnosis and treatment of FH vary when compared to adults^22^. Patients with a FH diagnosis prior to study entry date (1 January 1999) or with a prior diagnosis of other inherited lipid disorders were excluded.

### Clinical features

Clinical features incorporated into all machine-learning models are documented in Box 1. These were derived from known associations between these features and having FH from previous literature, in recommended diagnostic criteria, previously developed algorithms, or expert clinical opinion. We included a range of clinical features which could either increase or decrease the likelihood of FH.

Identifying patients with possible FH using Simon-Broome criteria is based on the following variables: total cholesterol, LDL-cholesterol, family history of MI, and family history of raised cholesterol^3^. Where a patients had both LDL and total cholesterol levels recorded, the LDL-cholesterol was prioritised, given its importance in recommended diagnostic criteria. For patients with multiple cholesterol levels recorded, the highest cholesterol value at any point between 1 January 1999 and 25 June 2019 was used. Each patient’s recorded triglyceride record at the time of each cholesterol measurement was extracted – raised levels are a negative predictor of FH^23^. We assessed for outliers (cholesterol and triglyceride levels ≤0 mmol/L or >5 positive standard deviations [SD] from the mean) and data entry errors. A prior history of CHD <60 years may also lead to a higher likelihood of being diagnosed with FH^27^.

Family histories of MI and of raised cholesterol were included as likely diagnostic variables^22^. while not specifically included in previous criteria/guidelines, family history of FH was also examined. Family history variables were dichotomised to either having a family history or not. In the event that family history was not evaluated, we assumed that there was no family history. Additional information was not available to further categorise family history to identify the relative affected and age at diagnosis for the condition^28^.

Although current diagnostic criteria use untreated cholesterol levels to evaluate probability of FH^27^, patients with elevated cholesterol levels may be receiving lipid-lowering therapy. Consequently, the prescribing and potency of lipid-lowering therapy were included as variables of interest. Cholesterol level was considered treated when the most recent prescription of lipid-lowering therapy ended within 30-days or overlapped with the date of the cholesterol measurement. A 30-day washout period was used to account for any residual effects of the lipid-lowering drugs when the drug treatment had been stopped^29^. Statin potency was classified using the most recent recommendations for statin intensity in the clinical guidance of the UK National Institute of Health and Care Excellence (NICE), which is based on a previous meta-analysis^30^.

Secondary causes of hypercholesterolaemia are currently recommended as negative predictors of FH in clinical guidelines^22^. The following important secondary conditions were, therefore, included in our assessment: liver disease (defined as, fatty liver disease, cirrhosis, chronic liver failure and alcoholic liver disease), diabetes mellitus (type I and type II), hypothyroidism (acquired and congenital), kidney disease (defined as, chronic kidney disease, renal impairment and acute renal failure) and nephrotic syndrome.

Box 1 Baseline predictor variables included in predicting familial hypercholesterolaemia

- Sex (female; male)
- Tendon xanthomata (yes; no)
- Family history of Familial Hypercholesterolaemia (yes; no)
- Family history of coronary heart disease, excluding myocardial infarction (yes; no)
- Family history of myocardial infarction (yes; no)
- Family history of raised cholesterol (yes; no)
- Family history of all coronary heart disease (yes; no)
- DNA test for apoB-100 (identified; not identified/no test)
- Any diagnosis of hypertension ever (yes; no)
- Any diagnosis of nephrotic syndrome ever (yes; no)
- Any diagnosis of coronary heart disease ever (yes; no)
- Any diagnosis of cerebrovascular accident ever (yes; no)
- Any diagnosis of peripheral vascular disease ever (yes; no)
- Any diagnosis of kidney disease ever (yes; no)
- Any diagnosis of hypothyroidism ever (yes; no)
- Any diagnosis of diabetes ever (yes; no)
- Any diagnosis of liver disease ever (yes; no)
- Most recent smoking status (non-smoker; ex-smoker; current smoker)
- Most recent alcohol status (non-drinker; ex-drinker; drinks)
- Most recent alcohol consumption (units/week)
- Highest potency statin ever prescribed (no statin usage recorded; other lipid-lowering drugs; low potency statins; medium potency statins; high potency statins)
- Highest total cholesterol level ever recorded (mmol/L)
- Age at time of highest total cholesterol record (years)
- Whether high total cholesterol was treated (treated; untreated)
- Treatment for high total cholesterol (untreated; other lipid-lowering treatment; low potency statins; medium potency statins; high potency statins)
- Triglyceride level at the time of highest total cholesterol record (mmol/L)
- Diastolic blood pressure closest to time of highest total cholesterol record (mmHg)
- Systolic blood pressure closest to time of highest total cholesterol record (mmHg)
- Hypertension control at the time of highest total cholesterol record (no hypertension; hypertension - unknown control; hypertension - poor control)
- Hypothyroidism control at the time of highest total cholesterol record (no hypothyroidism; hypothyroidism - unknown control; hypothyroidism - poor control)
- Diabetes control at the time of highest total cholesterol record (no diabetes; diabetes - unknown control; diabetes - poor control)
- Liver damage at the time of highest total cholesterol record (no liver disease; liver disease - unknown control; liver disease - poor control)
- Kidney disease at the time of highest total cholesterol record (no kidney disease; kidney disease - unknown control; kidney disease - poor control)
- Highest LDL-cholesterol level ever recorded (mmol/L)
- Age at time of LDL-cholesterol measurement (years)
- Whether high LDL-cholesterol was treated (treated; untreated)
- Treatment for high LDL-cholesterol (untreated; other lipid-lowering treatment; low potency statins; medium potency statins; high potency statins)
- Triglyceride level at the time of highest LDL-cholesterol record (mmol/L)
- Diastolic blood pressure closest to time of highest LDL-cholesterol record
- Systolic blood pressure closest to time of highest LDL-cholesterol record
- Hypertension control at the time of highest LDL-cholesterol record (no hypertension; hypertension - unknown control; hypertension - poor control)
- Hypothyroidism control at the time of highest LDL-cholesterol record (no hypothyroidism; Hypothyroidism - unknown control; Hypothyroidism - poor control)
- Diabetes control at the time of highest LDL-cholesterol record (no diabetes; diabetes - unknown control; diabetes - poor control)
- Liver damage at the time of highest LDL-cholesterol record (no liver disease; liver disease - unknown control; liver disease - poor control)
- Kidney disease at the time of highest LDL-cholesterol record (no kidney disease; kidney disease - unknown control; kidney disease - poor control)

### Outcome

The primary outcome was a documented incident diagnosis of FH in the patient records during the specified study period. FH is explicitly coded using the internationally recognised Read coding system in UK primary electronic health records (EHRs). This diagnostic code is entered into primary care electronic records after lipid specialist assessment, based on clinical phenotype, and/or by genetic test. To ensure temporality between predictors and the outcome, the diagnosis of FH must have occurred after the predictor variables.

### Machine-learning algorithms/models

The total study cohort was randomly split into a ‘training’ cohort (75% of the study cohort) in which the FH algorithms were derived and a ‘validation’ cohort (remaining 25% of the cohort) in which the algorithms were applied and tested. The data split was computer-generated using a uniform distribution to generate random numbers in STATA. The five commonly used algorithms were used – logistic regression^31^, random forest^32^, gradient boosting machines^33^, deep-learning neural networks^34^ and ensemble learning^35^. Ensemble learning model was a combination of the four (4) other ML algorithms. Using the library package h2o (http://www.h2o.ai) in R Studio, the risk algorithms were developed in the training cohort and applied to the validation cohort. A grid search was used to determine the hyper parameters for each model and 10-fold cross-validations was done to determine the values for the best performance using the training cohort (Supplementary Methods 1).

### Statistical analysis

Descriptive characteristics for the study population are reported as numbers with percentages or mean with standard deviation (SD) for categorical and continuous variables, respectively. The level of missing values ranged between 2.4% for systolic blood pressure to 23.3% for body mass index (BMI) (Supplementary Methods 2). To estimate missing values for BMI, LDL-C levels, triglyceride levels, systolic and diastolic blood pressures, multiple imputation by chained equations was used to generate 10 imputed datasets using all the other available patient variables^36^. The imputed datasets were pooled into a single dataset using Rubin’s rule^37^.

Harrell’s c-statistic, a measure of the total area under the receiver operating characteristic curve (AUC), was calculated using the validation cohorts to determine the predictive accuracy of the models developed in the training cohort. A jack-knife procedure was used to estimate the standard errors and 95% confidence intervals for the c-statistic estimates^38^. AUC is a global indicator of a test’s ability to determine whether or not a specific condition is present^39^. AUC value lies between 0.5 and 1.0–0.5 indicates a poor classifier and 1.0 indicates an excellent classifier. Calibration, the degree of similarity between observed and predicted probability of sub-optimal response, was assessed by a calibration plot in groups across the risk spectrum as recommended in TRIPOD guidelines^40^. Sensitivity, specificity, positive predictive value, and negative predictive value were calculated using a probability threshold of >1 in 250 (0.004)^4^ to reflect the expected prevalence of FH in the general population. Stata 16 MP4 version was used for statistical analyses to assess model performance.

### Informed consent/IRB statement

Ethical approval for this study was obtained from the Independent Scientific Advisory Committee (ISAC) – study protocol number 19_083R. De-identified (anonymised) patient data was obtained from CPRD hence this study was exempt from obtaining informed consent from patients.

### Reporting summary

Further information on research design is available in the Nature Research Reporting Summary linked to this article.

## Supplementary information

Supplementary Information

Reporting Summary

## Data Availability

The data that support the findings of this study are available from Clinical Practice Research Datalink (CPRD), but restrictions apply to the availability of these data, which were used under license for the current study, and so are not publicly available. Data are however available from the authors upon reasonable request and with permission of CPRD.

## Code availability

Codes are available at: https://github.com/PRISM-UoN/PRISM—Familial-Hypercholesterolaemia-Supervised-Machine-Learning-Models.
