# Supplementary material for: Performance and clinical utility of supervised machine-learning approaches in detecting familial hypercholesterolaemia in primary care
Source: NPJ Digit Med. 2020 Oct 30;3:142. doi: 10.1038/s41746-020-00349-5 (PMC7603302; doi:10.1038/s41746-020-00349-5)
Supplement: Supplementary file 1 — Supplementary Information [file 41746_2020_349_MOESM1_ESM.pdf]

# SUPPLEMENTARY FILE

# Performance and Clinical Utility of Supervised Machine-Learning Approaches in Detecting Familial Hypercholesterolaemia in Primary Care

| | |
|---|---|
| Supplementary Box 1 | **Tools for identifying familial hypercholesterolaemia based on clinical characteristics** |
| Supplementary Table 1 | **Top 10 risk factors for familial hypercholesterolaemia** |
| Supplementary Table 2 | **Model discrimination in the validation cohort for identifying familial hypercholesterolaemia in primary care (n = 1,006,943)** |
| Supplementary Methods 1 | **Supervised machine learning models using H20 machine learning engine** |
| Supplementary Methods 2 | **Variables with missing values** |

**Supplementary Box 1 Tools for identifying familial hypercholesterolaemia based on clinical characteristics**

| Tool | Clinical characteristics |
|---|---|
| **Simon Broome diagnostic criteria** | • Age (years)<br>• Total cholesterol<br>• LDL cholesterol<br>• Tendon xanthomas – *Patient, $1^{st}$ degree relative, or $2^{nd}$ degree relative*<br>• DNA-based evidence of an LDL receptor mutation or familial defective apo B-100 or a PCSK9 mutation<br>• Family history of premature CVD events *Myocardial infarction <50 years (2nd degree relative) or <60 years (1st degree relative)*<br>• Family history of raised cholesterol *>289.6 mg/dL (7.5 mmol/L) in adult $1^{st}$ or $2^{nd}$ degree relative or >258.7 mg/dL (6.7 mmol/L) in a child or sibling <16 years* |
| **Dutch Lipid Clinic Network criteria** | • First-degree relative with known premature coronary and vascular disease OR First-degree relative with known LDL-C level above the 95th percentile<br>• First-degree relative with tendinous xanthomata and/or arcus cornealis OR Children aged less than 18 years with LDL-C level above the 95th percentile<br>• Patient with premature coronary artery disease<br>• Patient with premature cerebral or peripheral vascular disease<br>• Tendon xanthomata<br>• Arcus cornealis prior to age 45 years<br>• LDL cholesterol<br>• Functional mutation in the LDLR, apo B or PCSK9 gene |
| **Make Early Diagnosis to Prevent Early Deaths (MEDPED)** | • Age<br>• Closest degree relative with confirmed FH diagnosis<br>*$1^{st}$ degree; $2^{nd}$ degree; $3^{rd}$ degree*<br>• Total cholesterol |
| **Japanese Atherosclerosis Society (JAS) criteria** | • Hyper-LDL cholesterolaemia (LDL-C before treatment: 180 mg/dL or more)<br>• Tendon xanthoma (tendon xanthoma on the dorsal hands, elbows, and knees, or Achilles tendon thickening) or nodular xanthoma on the skin<br>• Family history within the $2^{nd}$ degree relatives FH or premature coronary artery disease |
| **Familial Hypercholesterolaemia Case Ascertainment Tool (FAMCAT)** | • Highest total cholesterol or LDL-cholesterol<br>• Age<br>• Triglyceride level during cholesterol measurement<br>• Lipid lowering drug usage during cholesterol measurement<br>• Family history of familial hypercholesterolaemia<br>• Family history of coronary heart disease<br>• Family history of raised cholesterol<br>• Diagnosis of diabetes mellitus<br>• Diagnosis of kidney disease |

**Supplementary Table 1 Top 10 risk factors for familial hypercholesterolaemia**
Algorithms were derived from training cohort of 3,020,832 patients

| Logistic regression model | Random Forest model | Gradient Boosting model | Deep learning model |
|---|---|---|---|
| Statin potency at baseline | Systolic BP at highest LDL-C | Systolic BP at highest LDL-C | Hypothyroidism control at highest LDL-C |
| Highest TC ever | Body mass index | Body mass index | Sex |
| Highest LDL-C ever | Triglyceride at highest LDL-C | Triglyceride at highest TC | Kidney disease at highest LDL-C |
| | Triglyceride at highest TC | Triglyceride at highest LDL-C | Family history of premature CHD |
| | Family history of FH | Age at highest LDL-C | Any diagnosis of diabetes ever |
| | Statin potency at baseline | Highest LDL-C ever | Tendon xanthomata |
| | Age at highest LDL-C | Age at highest TC | Hypertension control at highest TC |
| | Age at highest TC | Statin potency at baseline | Family history of all CHD |
| | Highest LDL-C ever | Family history of FH | Liver disease at highest TC |
| | Highest TC ever | Highest TC ever | Any diagnosis of CHD ever |

**Supplementary Table 2 Discrimination accuracy of the models for identifying familial hypercholesterolaemia in primary care using the validation cohort (n = 1,006,943)**

| Models | AUROC (c-statistic) | Standard Error | 95% Confidence Interval | Absolute change |
|---|---|---|---|---|
| **Logistic regression model** | 0.8115 | 0.005 | 0.8008 – 0.8223 | - |
| **Random forest model** | 0.8909 | 0.004 | 0.8827 – 0.8991 | +7.94% |
| **Gradient boosting model** | 0.8919 | 0.004 | 0.8838 – 0.8998 | +8.04% |
| **Deep-learning model** | 0.8917 | 0.004 | 0.8836 – 0.8998 | +8.02% |
| **Ensemble learning model** | 0.8904 | 0.004 | 0.8822 – 0.8987 | +7.89% |

Jack-knife procedure was used to estimate standard errors

**Supplementary Methods 1 Supervised machine learning models using H20 machine learning engine**

Development of the risk algorithms in the training cohort and application of the risk algorithms to the validation cohort was completed using H20 (http://www.h20.ai). H2O is open-source software for big-data analysis. It allows users to fit thousands of potential models as part of discovering patterns in data. Five (5) different supervised machine learning models (Logistic Regression, Random Forest, Gradient Boosting Machine, Neural Network, and Ensemble Model) were built using H2O in R.

## Logistic regression

The common technique used in medicine for binary outcomes is logistic regression[1]. This is a generalised linear model that models the log odds of the dependant variable (class) as a linear combination of the independent variables (features),

$$\log\left(\frac{p_i}{1-p_i}\right) = \beta_0 + \beta_1 x_{1i} + \cdots + \beta_m x_{mi}$$

where the maximum likelihood is used to find the parameters that are most likely given the observed data. It is standard to use a zero mean normal prior for coefficients for regularisation.

Hyper-parameters:

nfolds = 10; fold_assignment = "AUTO"; keep_cross_validation_predictions = TRUE; family = "binomial"; alpha = 0.144; lambda = 0.005041

The optimal supervised learning model depends on the problem domain. There is no model that consistently outperforms other models for all problems. Consequently, to reduce the variability of models, it is often desirable to combine lots of diverse models. This is known as ensemble learning. There are three population ensembles: bagging, boosting and stacking[2]. Bagging involves creating numerous diverse models by randomly partitioning the training set (i.e., randomly selecting a subset of training examples or randomly selecting a subset of features) and often combining the models using majority voting. An example of a bagging ensemble is random forest[3]. Boosting is another way to combine models, but unlike bagging where the model are created independently, boosting adds models to an existing set of models based on the new model performing well at predicting the outcome for training examples that the existing models in the ensemble performed poorly on. An example of a boosting algorithm is gradient boosting machines[4]. Boosting can reduce the variance and bias of a model[5] but is susceptible to

noise[6]. As the class labels in this research have been identified using routine primary care data, there should be little noise in the labels.

**Random forest**

The random forest algorithm[3] averages multiple diverse decision tree predictions. Formally, the algorithm involves learning *ntree* diverse decision trees, $f_i(x): X \rightarrow Y$ where **X** is the input variables and Y is the outcome, each trained using a different random selection (with replacement) of *mtry* variables. The overall prediction then takes the majority voting of all *ntree* decision trees,

$$(x) = \begin{cases} 1, & if \frac{\sum_i f_i(\boldsymbol{x})}{ntree} > 0.5 \\ 0, & otherwise \end{cases}$$

The hyper-parameters of the random forest control the complexity of the learning function. A random forest with a high max depth (maximum number of interactions between independent variables) and high *mtry* (number of variables included in each tree) is more complex and thus, more likely to cause over-fitting. Therefore, limiting the max depth or *mtry* can effectively perform regularisation and reduce the chance of overfitting. This can also be accomplished by sampling the number of data points to be used for each tree (row sample).

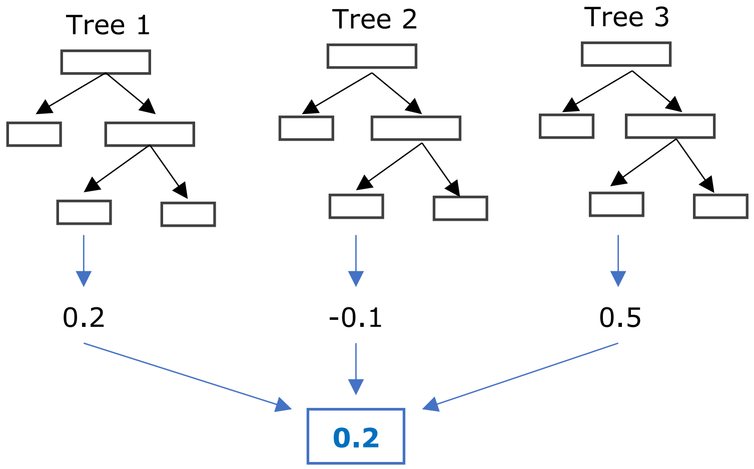


Hyper-parameter:

nfolds = 10; fold_assignment = "AUTO"; keep_cross_validation_predictions = TRUE; ntrees = 10000; max_depth = 13; min_rows = 10; nbins = 30; nbins_cats = 256; mtries = -1; sample_rate =0.7; stopping_metric = "AUC"; stopping_tolerance = 0.001; stopping_rounds = 2

### Gradient boosting machines

Gradient boosting machines[4] aim to minimise the loss function (a measure of difference between the observed and predicted values) by combining a sequence of base-learner models. A common optimisation method to find a minimum is gradient decent which involves going down a gradient to reach a minimum. The key idea behind gradient boosting machines is to sequentially add a new base learner model to the ensemble sequence such that the new model is the model with the greatest correlation with the negative of the loss function's gradient calculated using the current ensemble sequence predictions.

The gradient boosting machines algorithm[4] is a boosting algorithm that sequentially combines decision trees such that each additional tree is trained with more weighting placed on correctly predicting data-points that the previous decision trees misclassified. In simple terms, each new tree aims to correct for the mistakes of the previous trees. Gradient boosting machines have been successfully implemented across a range of classification tasks but are known to have performance issues when there is noise present in the data.

The hyper-parameters of the gradient boosting machine control the complexity of the learning function. A gradient boosting machine with a high max depth (maximum number of interactions between independent variables), high *ntree* (number of trees) and low observations per node (minimum number of data points required for each end node) is more complex and thus, more prone to overfitting. Therefore, limiting the max depth, *ntree* or increasing the observations per node can effectively perform regularisation and reduce the chance of overfitting.

Hyper-parameters:

> nfolds = 10; fold_assignment = "AUTO"; keep_cross_validation_predictions = TRUE; ntrees = 10000; max_depth = 11; min_rows = 100; nbins = 300; nbins_cats = 256; learn_rate = 0.01; learn_rate_annealing =0.999; sample_rate = 0.7; col_sample_rate = 0.7; stopping_metric = "AUC"; stopping_tolerance = 0.001; stopping_rounds = 2

### Neural network

A neural network[7] is a supervised learning algorithm consisting of a network with an input layer, hidden layers and output layer. The output of each non-input layer node consists of a weighted linear combination of the previous layer nodes' outputs transformed by a non-linear function. This is illustrated in the figure below:

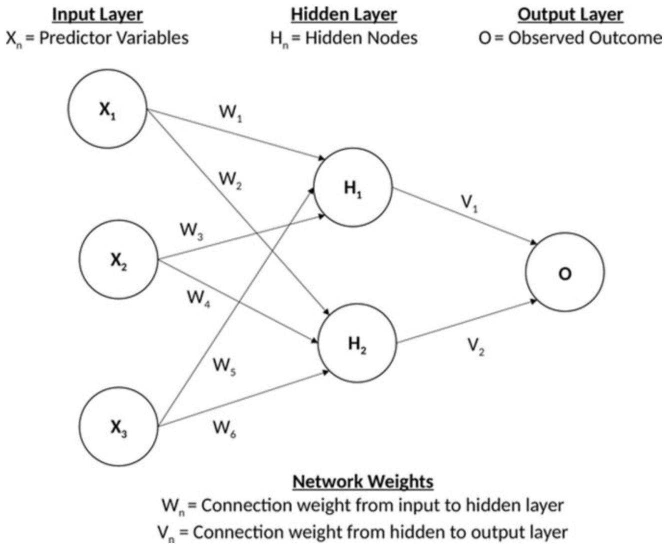


The multiple hidden layer neural network has been shown to be a universal approximation[8], meaning it is suitable for modelling any function. For the neural networks, the size of the network (number of hidden nodes) controls the complexity of the model whereas the decay performs regularisation.

Hyper-parameters:

> nfolds = 10; fold_assignment = "AUTO"; keep_cross_validation_predictions = TRUE; activation = "Maxout"; hidden = c(200, 200, 200); epochs = 200; adaptive_rate = FALSE; rho = 0.999; epsilon = 1e-06; rate = 0.001; rate_annealing = 1e-6; momentum_start = 0; momentum_stable = 0.99;
>
> input_dropout_ratio = 0.2; l1 = 1e-5; l2 = 1e-4; stopping_metric = "AUC"; stopping_tolerance = 0.001; stopping_rounds = 2

**Ensemble Model**

Ensemble methods use multiple learning algorithms to obtain better predictive performance. A meta-model then aggregates the predictions of individual models and outputs a final prediction. The final prediction is more robust and less prone to errors than each individual model as shown below:

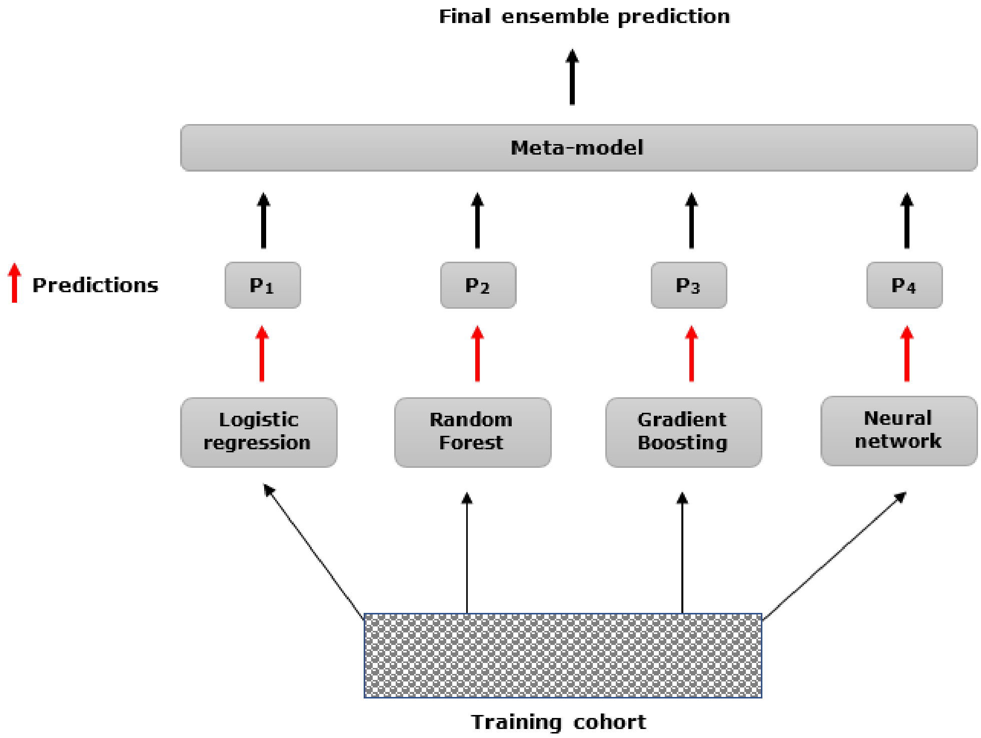


## References


1. Hosmer DW, Lemeshow S, Sturdivant RX. Applied Logistic Regression, 3rd Edition. New Jersey, USA: John Wiley & Sons; 2013.
2. Valentini G, Masulli F. Ensembles of Learning Machines. In: Marinaro M, Tagliaferri R, eds. Neural Nets: 13th Italian Workshop on Neural Nets, WIRN VIETRI 2002 Vietri sul Mare, Italy, May 30 – June 1, 2002 Revised Papers. Berlin, Heidelberg: Springer Berlin Heidelberg; 2002: 3-20.
3. Breiman L. Random Forests. Machine Learning 2001; 45(1): 5-32.
4. Friedman J. Greedy boosting approximation: a gradient boosting machine. The Annals of Statistics 2001; 29(5): 1189-232.
5. Freund Y, RE S. Experiments with a new boosting algorithm. ICML 1996; 96.
6. Dietterich TG. An Experimental Comparison of Three Methods for Constructing Ensembles of Decision Trees: Bagging, Boosting, and Randomization. Machine Learning 2000; 40(2): 139-57.
7. Hagan M, Demuth H, Beale M, De Jesus O. Neural Network Design, 2nd Edition. Boston: PWS Publishers; 2014.
8. Hornik K, Stinchcombe M, White H. Multilayer feedforward networks are universal approximators. Neural Networks 1989; 2(5): 359-66.

**Supplementary Methods 2 Variables with missing values**

| Variables | Number with missing value | Percent missing |
|---|---|---|
| Body mass index (BMI) | 937,987 | 23.29 |
| Highest LDL-cholesterol level ever recorded | 857,186 | 21.28 |
| Triglyceride level at time of highest total cholesterol record | 618,101 | 15.35 |
| Triglyceride level at time of highest LDL-cholesterol record | 617,495 | 15.33 |
| Diastolic blood pressure at time of highest total cholesterol record | 96,871 | 2.41 |
| Systolic blood pressure at time of highest total cholesterol record | 96,021 | 2.38 |
| Diastolic blood pressure at time of highest LDL-cholesterol record | 96,349 | 2.39 |
| Systolic blood pressure at time of highest LDL-cholesterol record | 95,797 | 2.38 |
