# Supplementary material for: Performance and clinical utility of supervised machine-learning approaches in detecting familial hypercholesterolaemia in primary care
Source: NPJ Digit Med. 2020 Oct 30;3:142. doi: 10.1038/s41746-020-00349-5 (PMC7603302; doi:10.1038/s41746-020-00349-5)
Supplement: Supplementary file 2 — Reporting Summary [file 41746_2020_349_MOESM2_ESM.pdf]

nature research

Corresponding author(s): DBPR NPJDIGITALMED-01059R

Last updated by author(s): Sep 14, 2020

Reporting Summary

Nature Research wishes to improve the reproducibility of the work that we publish. This form provides structure for consistency and transparency in reporting. For further information on Nature Research policies, see our Editorial Policies and the Editorial Policy Checklist.

Statistics

For all statistical analyses, confirm that the following items are present in the figure legend, table legend, main text, or Methods section.

n/a	Confirmed	
☒	☐	The exact sample size (n) for each experimental group/condition, given as a discrete number and unit of measurement
☒	☐	A statement on whether measurements were taken from distinct samples or whether the same sample was measured repeatedly
☐	☒	The statistical test(s) used AND whether they are one- or two-sided<br>*Only common tests should be described solely by name; describe more complex techniques in the Methods section.*
☐	☒	A description of all covariates tested
☒	☐	A description of any assumptions or corrections, such as tests of normality and adjustment for multiple comparisons
☐	☒	A full description of the statistical parameters including central tendency (e.g. means) or other basic estimates (e.g. regression coefficient) AND variation (e.g. standard deviation) or associated estimates of uncertainty (e.g. confidence intervals)
☒	☐	For null hypothesis testing, the test statistic (e.g. F, t, r) with confidence intervals, effect sizes, degrees of freedom and P value noted<br>*Give P values as exact values whenever suitable.*
☒	☐	For Bayesian analysis, information on the choice of priors and Markov chain Monte Carlo settings
☒	☐	For hierarchical and complex designs, identification of the appropriate level for tests and full reporting of outcomes
☒	☐	Estimates of effect sizes (e.g. Cohen's d, Pearson's r), indicating how they were calculated

Our web collection on statistics for biologists contains articles on many of the points above.

Software and code

Policy information about availability of computer code

Data collection	Electronic health record obtained from the Clinical Practice Research Datalink (CPRD).<br>Not data collection tool was used for this study.
Data analysis	Stata 16 MP4 version<br>R Studio

For manuscripts utilizing custom algorithms or software that are central to the research but not yet described in published literature, software must be made available to editors and reviewers. We strongly encourage code deposition in a community repository (e.g. GitHub). See the Nature Research guidelines for submitting code & software for further information.

Data

Policy information about availability of data

All manuscripts must include a data availability statement. This statement should provide the following information, where applicable:

- Accession codes, unique identifiers, or web links for publicly available datasets
- A list of figures that have associated raw data
- A description of any restrictions on data availability

The data that support the findings of this study are available from Clinical Practice Research Datalink (CPRD), but restrictions apply to the availability of these data, which were used under license for the current study, and so are not publicly available. Data are however available from the authors upon reasonable request and with permission of CPRD.

Field-specific reporting

Please select the one below that is the best fit for your research. If you are not sure, read the appropriate sections before making your selection.

☐ Life sciences ☒ Behavioural & social sciences ☐ Ecological, evolutionary & environmental sciences

For a reference copy of the document with all sections, see nature.com/documents/nr-reporting-summary-flat.pdf

Behavioural & social sciences study design

All studies must disclose on these points even when the disclosure is negative.

Study description	Retrospective cohort study using routine primary care clinical records
Research sample	The study cohort was obtained from the Clinical Practice Research Datalink (CPRD). CPRD contains anonymised electronic medical records from 836 general practices with over 11 million research-usable patients 21 and is representative of the UK general population.22 Over 5.2 million of these patient records are currently of active research-quality, making CPRD one of the most widely used real-world data sources for healthcare research.
Sampling strategy	No sample size calculation was performed.
Data collection	Not applicable
Timing	1 January 1999 to 25 June 2019
Data exclusions	Patients aged 16 years and younger were excluded as the cholesterol level thresholds for the diagnosis and treatment of FH vary when compared to adults.23 Patients with a FH diagnosis prior to study entry date (1 January 1999) or with a prior diagnosis of other inherited lipid disorders were excluded.
Non-participation	Not applicable
Randomization	The total study cohort was randomly split into a 'training' cohort (75% of the study cohort) in which the FH algorithms were derived and a 'validation' cohort (remaining 25% of the cohort) in which the algorithms were applied and tested. The data split was computer-generated using a uniform distribution to generate random numbers in STATA.

Reporting for specific materials, systems and methods

We require information from authors about some types of materials, experimental systems and methods used in many studies. Here, indicate whether each material, system or method listed is relevant to your study. If you are not sure if a list item applies to your research, read the appropriate section before selecting a response.

Materials & experimental systems

n/a	Involved in the study	
☒	☐	Antibodies
☒	☐	Eukaryotic cell lines
☒	☐	Palaeontology and archaeology
☒	☐	Animals and other organisms
☐	☒	Human research participants
☐	☒	Clinical data
☒	☐	Dual use research of concern

Methods

n/a	Involved in the study	
☒	☐	ChIP-seq
☒	☐	Flow cytometry
☒	☐	MRI-based neuroimaging

Human research participants

Policy information about studies involving human research participants

Population characteristics	Patients with a record of cholesterol level is essential to establish a diagnosis of FH hence, all patients included in the study had at least a single record of total cholesterol measurement between the baseline date of 1 January 1999 or the earliest date the CPRD primary care practice started contributing data to the database after 1 January 1999 and the end date of 25 June 2019 or the latest date the CPRD primary care practice finished contributing data prior to 25 June 2019.
Recruitment	Participants were identified retrospectively using electronic health records.
Ethics oversight	Ethical approval for this study was obtained from the Independent Scientific Advisory Committee (ISAC) – study protocol number 19_083R.

Note that full information on the approval of the study protocol must also be provided in the manuscript.

Clinical data

Policy information about clinical studies

All manuscripts should comply with the ICMJE guidelines for publication of clinical research and a completed CONSORT checklist must be included with all submissions.

Clinical trial registration	Not applicable
Study protocol	Not applicable
Data collection	Not applicable
Outcomes	Not applicable
